# Co‐Designing a Fall Prevention Exercise Website to Meet Aged Care Community Needs

**DOI:** 10.1111/ajag.70216

**Published:** 2026-08-07

**Authors:** Rik Dawson, Lauren Cameron, Marin B. Pinheiro, Katrina Jarratt, Catherine Sherrington, Abby Haynes

**Affiliations:** ^1^ The University of Sydney Institute for Musculoskeletal Health, Sydney Local Health District Sydney Australia; ^2^ University of New South Wales Sydney Australia

**Keywords:** age friendly health care, community‐based participatory research, digital health, falls, user centred design

## Abstract

**Objectives:**

Falls among older people are a significant health issue. Exercise reduces falls, yet many older people do not meet physical activity guidelines. Digital health platforms may improve access to fall prevention programs, but limited research has explored how these platforms meet older people's needs. This study aimed to use co‐design principles to develop a website supporting delivery of the TOP UP program in aged care. A secondary aim explored older people's experiences of participating in the co‐design process.

**Methods:**

A three‐phase co‐design study was conducted. Phase 1 used interviews, focus groups and heuristic evaluation to inform development of a prototype website. Participants included 14 older people receiving aged care services (median age 84 years), 10 care staff and six physiotherapists. Phase 2 used think‐aloud usability interviews with 11 older people, including Likert scale ratings, to assess navigation, clarity and functionality and refine the prototype. Phase 3 explored participants' experiences of the co‐design process.

**Results:**

Thematic analysis identified eight design themes highlighting priorities related to simplicity, accessibility, relatability, safety, customisation and engaging features that supported motivation while minimising staff burden. Feedback on the website was positive and informed improvements in readability, navigation and credibility. Participants reported positive co‐design experiences and valued contributing to the development of a digital fall prevention resource.

**Conclusions:**

Co‐design is valuable in developing user‐centred digital health solutions for aged care. Findings highlight the importance of aligning digital platforms with older people's needs to improve accessibility, engagement and scalability, supporting implementation of programs such as TOP UP.

## Impact Statements

The TOP UP program leverages telephysiotherapy to enhance mobility and reduce falls in aged care. Co‐designed with stakeholders, it improves accessibility through an intuitive website. This study highlights the importance of simplicity, relatability, capability customisation and user engagement in digital health solutions, ensuring effective, scalable and inclusive aged care interventions.

## 1 Introduction

Falls among Australians aged 65 years and older cost the healthcare system A$2.8 billion annually, with nearly half of aged care residents experiencing a fall each year [1, 2]. Falls are often linked to impaired physical or cognitive function [3]. Exercise reduces falls by 23% in community‐dwelling older adults, particularly when balance and functional movements, such as sit to stand, are targeted [4]. The World Health Organization recommends these exercise approaches, yet access to evidence‐based, physiotherapist‐led exercise programs and restorative care services remains limited, especially in rural and underserved aged care settings, as highlighted by the Australian Royal Commission into Aged Care Quality and Safety [5, 6].

**FIGURE 1:**
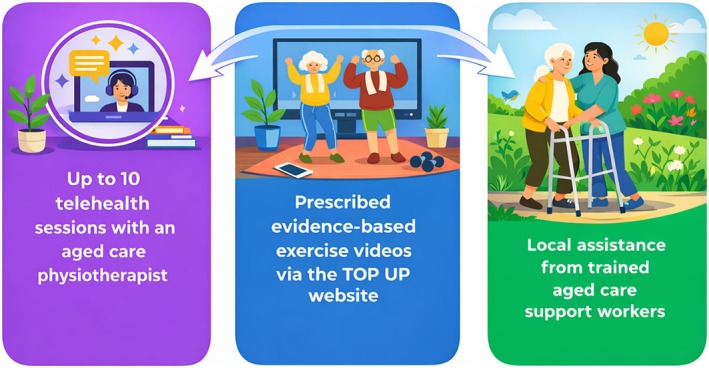
Key elements of the TOP UP program.

The TOP UP program was developed to address this gap by connecting older people receiving aged care services with physiotherapists through digital platforms such as Zoom and a dedicated website delivering moderate intensity balance and strength exercises designed to improve mobility, prevent falls and enhance quality of life (Figure 1). The program was evaluated in the TOP UP trial, a six‐month hybrid Type 1 effectiveness‐implementation randomised controlled trial conducted between September 2021 and December 2023 [7]. The trial tested synchronous telephysiotherapy via Zoom alongside asynchronous support through exercise videos with assistance from care staff. A total of 242 older people receiving aged care services were recruited.

The trial not only demonstrated feasibility and positive impact but also highlighted the need for a more user‐friendly website to better integrate program components and improve accessibility and engagement [8]. Qualitative analysis suggested that the acceptability of TOP UP is driven by telephysiotherapy expanding access to care and supporting engagement through tailored guidance, therapeutic alliance and capability‐appropriate resources that enable independent exercise [8].

To maximise engagement, accessibility and impact of digital health programs such as TOP UP, the literature recommends that supporting websites align with the needs and capabilities of older people, physiotherapists and care staff [9]. Kebede et al. [9] described key barriers to digital engagement, including capability (e.g., physical and cognitive changes), opportunity (e.g., access to and usability of technology) and motivation (e.g., perceived usefulness). Addressing these barriers requires user‐centred design approaches that prioritise intuitive interfaces, accessible content and meaningful functionality [10].

Co‐design integrates stakeholders' perspectives throughout the design process, from problem identification to solution testing [11]. This participatory approach enables researchers to better understand user needs while supporting the development of practical and acceptable solutions [11]. The primary aim of this study was to apply co‐design principles to develop and test a website to enhance delivery of the TOP UP program in aged care. A secondary aim was to understand how older people experienced the co‐design process.

## 2 Method

### 2.1 Study Design

**FIGURE 2:**
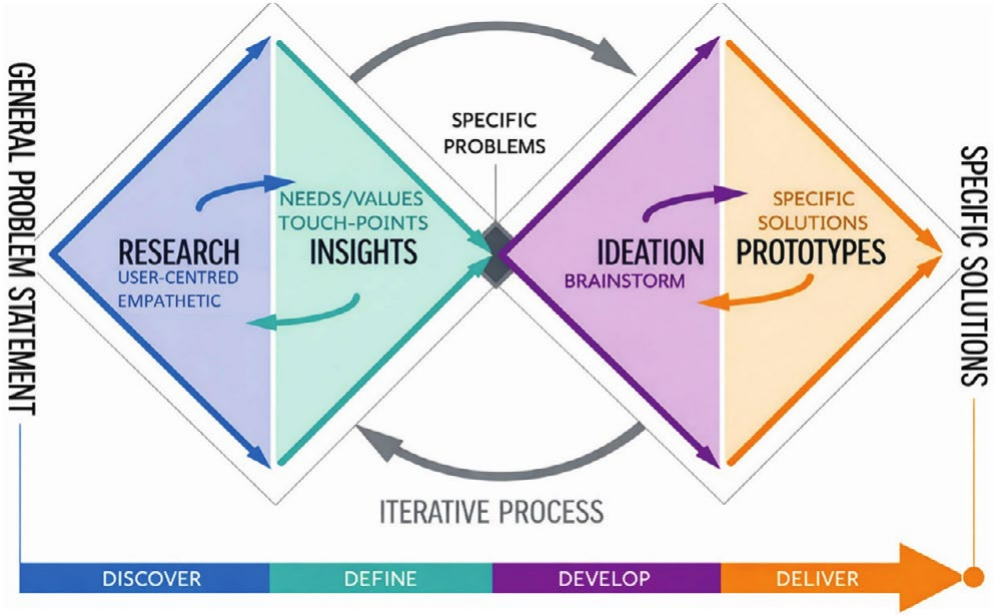
The ‘Double Diamond’ model of co‐design [12].

This study utilised a three‐phase qualitative design guided by the Double Diamond model of co‐design, a user‐centred approach commonly used in digital health research with older people [12]. The Double Diamond process systematically explores and refines solutions through four stages: *Discover*, where problems are identified, insights are gathered, and user needs are explored; *Define*, where problems are articulated and refined to provide actionable guidance; *Develop*, where solutions are collaboratively generated and explored; and *Deliver*, where solutions are tested, evaluated, and iteratively improved by retaining effective elements and discarding ineffective ones (Figure 2).

The core of this model is the development of a shared problem statement to ensure researchers and participants have a mutual understanding of the research objectives [13]. The problem statement for this study was derived from formative qualitative research conducted with participants, physiotherapists and care staff during the pilot trial [8]:

Service users can benefit from participation in the TOP UP program, but the current website on which the program depends is hard to use and does not incorporate all the program components (e.g., recording exercise activities). This can be confusing for all stakeholders, especially service users. It also creates additional time and technological demands for care staff and physiotherapists, potentially deterring their continued engagement with the program.

### 2.2 Recruitment and Consent

Stakeholders from residential and home‐based aged care services involved in the TOP UP trial in New South Wales, Australia, were invited to participate. Aged care partners first obtained verbal consent for the research team to contact potential participants identified from trial records. Potential participants were then contacted by phone by members of the research team (R.D. or L.C.) and provided an information sheet and consent form via email. Written consent was obtained prior to participation. Purposive sampling was used to maximise diversity among older people. Sample sizes were consistent with qualitative co‐design research, and data collection continued until feedback became repetitive and minimal new usability issues were identified [14, 15]. There was no recruitment incentive offered.

### 2.3 Data Collection

#### 2.3.1 Phase 1: Co‐Designing the TOP UP Website

Phase 1 sought to *discover* and *define* challenges with the existing TOP UP website and its role in program delivery. Semi‐structured interviews and focus groups were conducted face‐to‐face or via Zoom with older people, physiotherapists and care staff. Interviews with older people were conducted in their homes, with local care staff assisting with technology where needed, while care staff and physiotherapists were interviewed at their workplaces. Interview guides were developed with input from the research team and aged care partners and informed by usability principles (Data S1: Appendix 1) [16]. Participants were asked to critique early ideas for the revised website generated from prior qualitative work and literature highlighting the importance of clear, simple interfaces and trustworthy content in digital health programs for older people [8, 17].

#### 2.3.2 Phase 2: User Testing and Iteration of the TOP UP Website Prototype

Eight weeks after Phase 1, Phase 2 aimed to *develop* and *deliver* the prototype website using ‘think‐aloud’ interviews informed by constructs from the App Evaluation Model for Assessing mHealth Apps A‐MARS (Data S1: Appendix 2) [18]. Eleven older people from Phase 1 navigated the website on an iPad while verbalising their thoughts. Participants provided real‐time feedback on functionality, usability, aesthetics and content quality. Usability was assessed through task completion, discussion of challenges and short Likert‐scale ratings (0–5) capturing participants' perceptions of ease of use, confidence in the information and willingness to engage with the website. Feedback from these interviews informed iterative refinements to navigation, layout and clarity of the website. The website was developed in collaboration with a digital health development company.

#### 2.3.3 Phase 3: Evaluation of the Co‐Design Process

Following Phase 2, telephone interviews were conducted with all consenting older people (*n* = 11) to explore their experiences of the co‐design process. Interviews included open‐ended questions and focused on key dimensions of participation. This phase examined acceptability, perceived value and any challenges or benefits [19].

Two experienced qualitative researchers, R.D. and L.C., conducted data collection in Phases 1 and 2. One author, R.D., a male postdoctoral researcher with aged care physiotherapy experience, was known to some participants through prior involvement, while L.C., a female research assistant specialising in user experience, had no prior relationship. Both had qualitative research experience. Participants were informed of the researchers' roles in evaluating and improving the TOP UP program. Both researchers had a professional interest in improving access to physiotherapy and digital health for older people, and reflexive discussions were undertaken within the research team to minimise potential bias. To minimise social desirability bias, participants were assured responses were confidential, would not affect care or employment and were encouraged to provide both positive and critical feedback [20]. Phase 3 interviews were conducted by an independent qualitative researcher (A.H.) with no prior relationship to participants to further reduce social desirability bias.

All interviews and focus groups were audio‐recorded using Zoom transcription software. One author (R.D.) reviewed and corrected transcripts, and researchers recorded field notes to document usability issues and key observations. Transcripts were not returned to participants, and no visual recordings were made. No repeat interviews were conducted within phases, and all participants were invited to take part in Phases 1 and 2, with only older people participating in Phase 3.

### 2.4 Data Analysis

#### 2.4.1 Phase 1: Interview and Focus Group Data

Transcripts were analysed using a qualitative descriptive approach to identify patterns and develop themes that accurately reflected participants' views and experiences [21]. This method generates clear descriptive summaries suited to informing health practice and addressing barriers faced by vulnerable populations [22]. Preliminary coding and identification of emerging themes occurred concurrently with data collection, allowing the research team to reflect on early findings and refine interview prompts where appropriate. Two researchers (R.D. and A.H.) independently coded two transcripts inductively and agreed on an initial coding framework. Transcripts were managed and analysed in Microsoft Word using highlighting and comment functions. One researcher (R.D.) then applied this framework to the remaining transcripts, with emerging themes discussed within the research team to support consistency in interpretation.

#### 2.4.2 Phase 2: Think‐Aloud Interview Data

Framework analysis was used to systematically synthesise stakeholder feedback and identify key usability issues [23]. The analysis followed the stages of framework analysis: familiarisation with the data, coding, indexing, charting and mapping to support interpretation. One researcher (A.H.) summarised relevant information from transcripts and field notes into a matrix based on a truncated version of the A‐MARS framework (e.g., user engagement, functionality, aesthetics, content quality and subjective quality) [18]. Transcripts were stored and analysed in Microsoft Word using highlighting and comment functions to code segments of text. Codes were compared across transcripts and grouped to identify patterns and develop themes. Themes from Phases 1 and 2 were then reviewed together and refined to identify overarching findings. Emerging results, supported by illustrative quotes, were discussed with the wider research team, who acted as ‘critical friends’ to provide alternative interpretations before operationalising results with the technology developer [24].

#### 2.4.3 Phase 3: Evaluation of the Co‐Design Process

Telephone interviews were analysed by one researcher (A.H.) using qualitative descriptive content analysis to identify patterns in participants' experiences of participating in the co‐design process [21].

### 2.5 Ethics

This project had ethics and governance approval by the Human Research Ethics Committee at The Sydney Local Health District, Concord, Australia (approval number CH62/6/2021‐009).

## 3 Results

Six physiotherapists, 10 support workers and 14 older people receiving aged care services in residential aged care or at home (median age 84 years; range 68–94) participated in the study. Table 1 outlines demographic characteristics. No invited participants declined participation. Eleven older people completed Phase 2 think‐aloud interviews and Phase 3 evaluations, with two withdrawing due to illness and one death. Interviews lasted 30–60 min. Data collection and analysis were conducted sequentially across the three study phases.

**TABLE 1:** Participant characteristics (*n* = 14).

Characteristic	Value
Age, years, median (range)	84 (68–94)
Sex, *n* (%)	
Women	10 (71)
Men	4 (29)
Living setting, *n* (%)	
Residential aged care	7 (50)
Home aged care	7 (50)
Mobility aid use, *n* (%)	
Nil	7 (50)
Walking frame	7 (50)
Cognitive impairment, *n* (%)	
Normal	11 (79)
Mild	3 (21)

**FIGURE 3:**
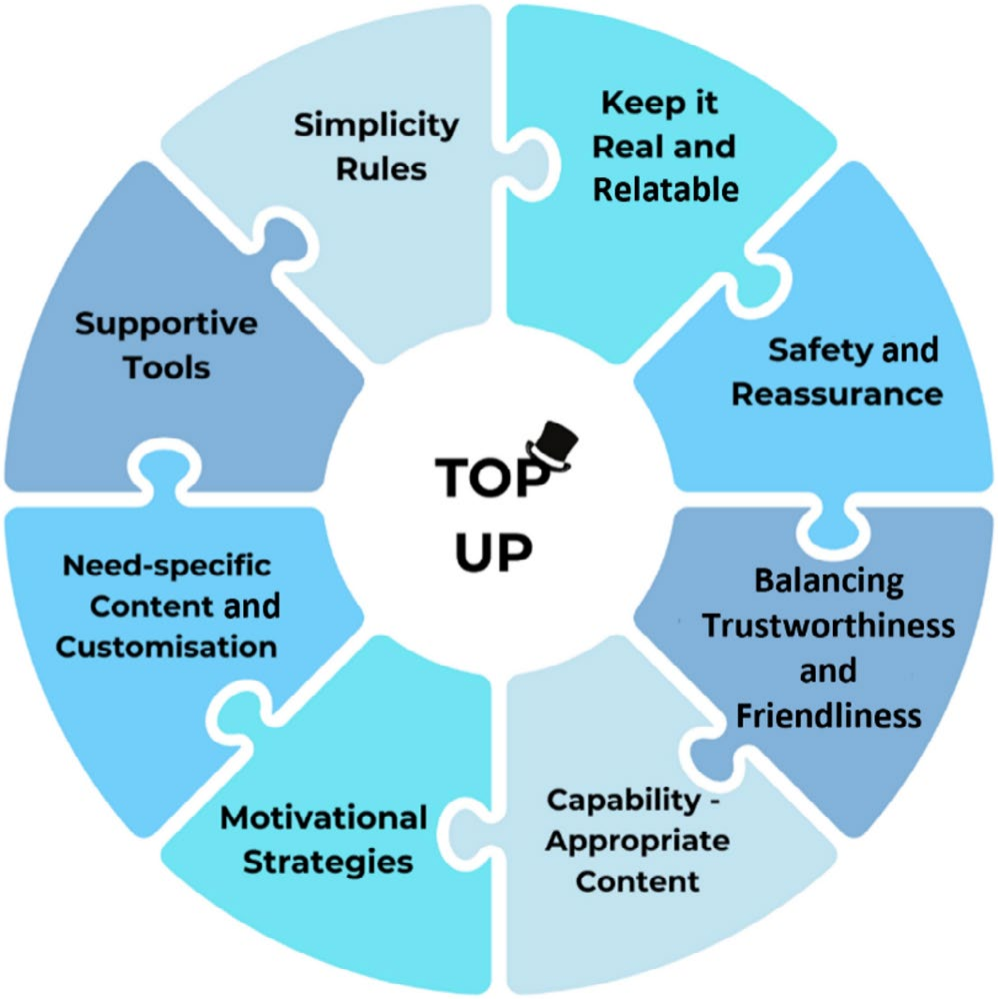
TOP UP co‐design thematic results.

To provide a coherent understanding of user experiences and website design priorities, findings from Phases 1 and 2 are presented thematically across phases rather than reported separately. Eight themes emerged from the analysis of interviews and think‐aloud sessions (Figure 3). Likert scale responses collected during Phase 2 evaluation are presented following the thematic findings to complement the qualitative results, with participant ratings of website usability and features summarised in Table 2.

**TABLE 2:** Think‐aloud results mapped transcripts to the α‐MARS domains [18].

A‐MARS construct	Participant quotes
Engagement	I think the website should have a friendly feeling, to put the client at ease. (aged care support worker)
2 Interest	I have seen some exercise apps where they use a young person explaining how to do a particular exercise and they just go zip, zip, zip and as an older person it takes a little more time to keep up, they have to realise that an older person may not be able to keep up. (older person)
3 Customisation	A different way for the future is [to] potentially allow an option that we can film an exercise ourselves and maybe incorporate that into their exercise plan. (physiotherapist)
4 Interactivity	I would like to be able to keep a track of my exercise record on the website as I feel I get a bit buried in the paperwork. (older person)
5 Target group suitability	I don't think that the program is suitable for people with severe dementia, they would need a lot of help, but the videos could be useful for the support staff to use. They could play the video and concentrate on encouraging the client to do the different exercises without having to worry about what is coming next. They are a great resource. (older person)
6 Performance	I feel more comfortable just clicking on one link to get the video. It would be great to click on a link on the website, and the physio is there ready to talk to. (older person)
7 Ease of use	I don't like that some apps need you to be always commenting how you are going, a simple way is better. (older person)
8 Navigation	I think it would be good to have the website menu in large font that spells out the type of exercise – like seated, balance and orthopaedic, it would be easy to direct the participants into the right program. (physiotherapist)
9 Layout	Make sure the website is not bunched up, make sure there is a lot of space, so it is easy to read, otherwise it turns you off. (older person)
10 Visual appeal	I like a standard white background nature with a little bit of colour. The black background with white writing seems harder to read. (physiotherapist)
11 Content quality	It was really good to have an older person demonstrating the exercises in the videos, because you could see exactly how it was done. The physio was good showing the exercise in the chair, but watching another elderly person, someone your own age, doing them gave you the incentive to do with them. (older person)
12 Comprehensiveness	I think some shorter videos that focus on a specific part of the body would be useful. (aged care support worker)
13 Credibility	I like the professional feel of the website. (older person)

### 3.1 Themes

#### 3.1.1 Simplicity Rules

A common theme across physiotherapists, support workers and older people was the need for a simple, intuitive design to encourage engagement, particularly among those unfamiliar with or intimidated by technology. Features such as clear navigation and single‐click options were favoured:

Get it as user‐friendly as possible. Well, the thing is, we didn't grow up with this technology – all the young people they've grown up with it, and it's just so simple for them. (older person)

Physiotherapists also emphasised the importance of simple navigation and clear exercise instructions to support safe independent practice between supervised sessions.

#### 3.1.2 Capability‐Appropriate Content

Physiotherapists emphasised the importance of clinically accurate exercise demonstrations and clear safety guidance for older people exercising independently between supervised sessions. Older people and support workers highlighted the need for content that accommodates diverse abilities, including large text, high contrast menus and uncluttered layouts to support users with visual impairments. Videos were preferred over text heavy content, particularly those with slow pacing, clear speech and exercises presented at progressive levels of difficulty to support varying physical abilities:

It's very easy on the eye, so easy to read, having the lighter background, having a separation of different types of exercises for the older person is also a good idea. Catering for different levels, and having different levels within each range of exercises, is also good. (aged care staff)

#### 3.1.3 Keep It Real and Relatable

Older people and support workers emphasised the importance of using authentic, relatable content to engage users and encourage participation, while physiotherapists highlighted the value of realistic demonstrations to support safe exercise practice. They expressed a preference for seeing real older people in photos and videos rather than stock images or cartoon characters, as this made the content feel more genuine and motivating. Many explained that watching individuals of similar age and ability performing exercises was more inspiring than seeing younger, more athletic people:

I think it's important to see someone in your age group in the videos. That's good and engaging. (older person)

#### 3.1.4 Motivational Strategies

Motivational elements, such as the encouraging tone in the videos and the exercise titles that acknowledge progress, were valued for sustaining user commitment without being prescriptive. Physiotherapists also noted that positive reinforcement embedded on the website could support adherence between supervised sessions. Online prompts and progress tracking offered encouragement. Service users desired simple tools to track their progress and receive reminders, such as digital exercise logs and automated notifications, to stay on track:

I would like to be able to keep track of my exercise in a simple way so I could show my physio what I have done. (older person)

#### 3.1.5 Safety and Reassurance

A consistent theme across physiotherapists, support workers and older people was the importance of clear safety instructions and demonstration videos illustrating correct exercise techniques to reduce risk and build confidence:

I like having the exercise video on the screen so I could see how to the exercise properly. I would like to see some videos on how to exercise safely at home. (older person)

#### 3.1.6 Balancing Friendliness and Trustworthiness

Participants highlighted the importance of balancing a professional, trustworthy appearance with a friendly atmosphere on the website. They preferred colour schemes featuring inviting tones such as sky blue, jade green and orange. While some users appreciated humour to create a light‐hearted and engaging environment, others preferred a more straightforward, factual approach that emphasised credibility and trust. The combination of warmth, professionalism and trust appeared to create a welcoming and engaging user experience:

Many clients find it hard to motivate themselves to exercise, so any program needs to have an engaging style, inject humour wherever you can. (aged care staff)

#### 3.1.7 Need‐Specific Content and Customisation

All three stakeholder groups wanted tailored exercise plans to address specific needs such as joint replacements and cognitive conditions like dementia. They also expressed a desire for flexibility to personalise exercise routines, including the option to upload tailored videos and adapt features to individual needs:

How about an option in the future where we can film exercises ourselves and incorporate them into a patient's exercise plan? We could then drag and drop exercises to personalise the program. (physiotherapist)

#### 3.1.8 Supporting Tech Transition

Support workers and physiotherapists highlighted the need for digital health tools that reduce staff workload. Features such as automated scheduling, instructional videos on session set‐up, and resources that empowered users to exercise independently were strongly advocated for. The revised TOP UP website was praised for its intuitive interface and practical support for aged care service users' independent exercise:

TOP UP is simple and familiar, intuitive, similar to typical internet navigation, which is appealing. It is another way of having a physiotherapist there with a client without them needing to be right there [physically]. It gives them [the client] autonomy to be able to do the sessions at a time that suits them. (physiotherapist)

To complement the thematic findings, Phase 2 interviews also included Likert‐scale ratings (0–5) of usability. The revised website received a score of 5 for ease of use and 4.5 for willingness to engage. Participants also identified areas for improvement, particularly navigation challenges for people with sensory impairments and the need for clearer video controls: *I need larger icons for play, pause, expand on videos. It is still hard to find these controls*. (older person).

The results of our framework analysis displayed in Table 2 show that the TOP UP website improvements align with key A‐MARS criteria [18]. Participants appreciated the friendly and welcoming design, clearer navigation and one‐click access for ease of use. The program's credibility is strong, developed with researchers, physiotherapists and aged care experts. Overall, revisions to the TOP UP website enhanced performance, engagement and accessibility, but further refinements in interactivity and customisation could optimise user experience.

Phase 3 analysis of the co‐design evaluation data indicated that some older people initially found it difficult to distinguish between their roles as TOP UP participants and co‐designers of the website. Despite this, all participants who commented on the co‐design process reported positive experiences. Many described the intellectual stimulation of participation and the satisfaction of making a meaningful contribution, seeing their suggestions implemented, and having a purposeful activity to focus on, which they felt was often lacking in everyday life. These findings suggest that involving older people in co‐design can strengthen the final product by ensuring it reflects their needs, preferences and lived experience. As one participant reflected:

I loved feeling that I was part of something intelligent and worthy … and almost still continuing with my education. It's stimulating … I love that feeling of being part of something. (older person)

## 4 Discussion

The findings of this study highlight the value of co‐design in developing digital health solutions to support exercise participation among older people. Input from older people, support workers and physiotherapists identified key priorities including simplicity, relatability and personalisation. Participants were satisfied with the revised website, particularly its ease of use and expanded content, although further refinements were suggested to support navigation for users with sensory impairments, include more relatable imagery and allow greater customisation. The Double Diamond model provided a structured approach to engaging end users and incorporating iterative feedback to improve usability and accessibility. These insights are also relevant for implementation, as digital fall prevention programs are more likely to be adopted and sustained when they align with the needs and capabilities of intended users.

The co‐design process was well‐accepted, with no invited stakeholders declining participation. Participants were recruited through aged care partners involved in the TOP UP trial, and many were familiar with the research team, which may have supported engagement through established trust. This level of participation suggests strong interest among older people and care staff in contributing to digital fall prevention interventions and demonstrates the feasibility of involving older people receiving aged care services in the design of digital health tools. Previous research has demonstrated that involving older people in co‐design can improve the usability, relevance and acceptability of digital health technologies [25].

These findings emphasise the importance of simple, intuitive website design for older people. User‐centred design has been shown to help bridge generational and technological gaps while addressing ageing‐related challenges [10, 26]. Participants emphasised the importance of clear instructions and engaging exercise content, intuitive navigation, personalised plans and relatable content, consistent with evidence that features such as progress tracking, goal setting and encouragement can support engagement in digital health programs [27]. Physiotherapists emphasised that the website needed to support safe exercise prescription while integrating easily into existing physiotherapy workflows within aged care services.

Future development of the TOP UP program will focus on improving navigation, expanding exercise content, maximising device compatibility and scaling the program to underserved areas. Features such as progress tracking, integration with activity monitors and digital tools for goal setting may support personalisation and long‐term engagement [27]. Translating content and ensuring compatibility across multiple devices may further enhance accessibility and inclusivity [28].

These findings also highlight opportunities to strengthen engagement and support implementation of digital exercise platforms in aged care settings. Features such as motivational prompts, progress badges and peer feedback may support behaviour change and sustained participation [29]. Expanding exercise content to include varied activities, such as dance, may further enhance engagement, particularly for individuals with cognitive impairment [30]. Implementation supports, including educational resources and technical assistance, will also be important for adoption within aged care services [31].

Positive feedback from older people about the co‐design process is consistent with evidence that participatory research can generate mutually beneficial outcomes for researchers and participants [32]. Co‐design approaches enable meaningful involvement of older people and service providers in shaping program development through community based participatory research [32].

A strength of this study is the structured co‐design approach, which engaged older people, care staff and physiotherapists in the development and refinement of the website. Involving end users throughout the process enabled identification of usability issues and ensured the platform reflected the needs and preferences of older adults receiving aged care services. The iterative three‐phase design, guided by the Double Diamond framework and incorporating interviews, focus groups and think‐aloud usability testing, strengthened the rigour of the development process and allowed participant feedback to directly inform website improvements.

Several limitations should be acknowledged. Data were collected from a single Australian state, and the semi‐structured think‐aloud task may not fully reflect natural user interactions. The participant group also lacked diversity, excluding service users with severe sensory or cognitive impairments and those from culturally and linguistically diverse backgrounds, which may limit the applicability of findings to these groups. Some participants were known to members of the research team through the TOP UP trial, which may have introduced social desirability bias. In addition, some transcripts were coded solely by the lead researcher, which may have introduced interpretation bias. Future work should involve further co‐design with service users, support workers and clinicians to refine the website and evaluate potential enhancements such as scaling the program, translating content, incorporating AI‐supported features and progress tracking to support feasibility and engagement.

## 5 Conclusions

The TOP UP program demonstrates the potential of co‐designed digital health platforms in aged care, offering scalable solutions to enhance mobility and prevent falls. Using the Double Diamond framework, this study showed the feasibility of user‐friendly, tailored digital interventions. Future research should explore large‐scale implementation, emphasising partnerships between researchers, healthcare providers and older people to optimise digital solutions for healthy ageing and independent living.

## Funding

R.D. received funding from the NHMRC‐funded Centre for Research Excellence—Prevention of Fall‐related Injuries. M.B.P. and C.S. received salary funding from National Health and Medical Research Council of Australia Fellowships. The TOP UP Study had funding from Dementia Australia and Aged Care Industry and Innovation Australia. This co‐design study was funded by a grant awarded to A.H. by the Australian Association of Gerontology. The funders had no role in the study design and will not have any role during its execution, analyses, interpretation of the data or decision to submit results.

## Ethics Statement

This study was granted ethical approval by the Human Research Ethics Committee at The Sydney Local Health District, Concord, Australia (approval number CH62/6/2021‐009).

## Conflicts of Interest

The authors declare no conflicts of interest.

## Supporting information


**Data S1:** ajag70216‐sup‐0001‐Supinfo1.docx.

## Data Availability

Data are available on reasonable request. Proposals for data should be directed to the corresponding author (rik.dawson@sydney.edu.au).

## References

[1] Australian Institute of Health and Welfare , “Falls,” (2024), https://www.aihw.gov.au/reports/injury/falls.

[2] I. D. Cameron , S. M. Dyer , C. E. Panagoda , et al., “Interventions for Preventing Falls in Older People in Care Facilities and Hospitals,” Cochrane Library 2020, no. 1 (2018): 4, 10.1002/14651858.CD005465.pub4. PMC6148705 30191554

[3] D. A. Ganz and N. K. Latham , “Prevention of Falls in Community‐Dwelling Older Adults,” New England Journal of Medicine 382, no. 8 (2020): 734–743, 10.1056/NEJMcp1903252. 32074420

[4] C. Sherrington , Z. A. Michaleff , N. Fairhall , et al., “Exercise to Prevent Falls in Older Adults: An Updated Systematic Review and Meta‐Analysis,” British Journal of Sports Medicine 51, no. 24 (2017): 1750–1758, 10.1136/bjsports-2016-096547. 27707740

[5] World Health Organization , “World Health Organization on Physical Activity and Sedentary Behaviour,” (2020), https://apps.who.int/iris/handle/10665/336656.

[6] “Aged Care Royal Commission's Final Report Calls for Care, Dignity and Respect,” InScope (2021), 20: 20.

[7] R. Dawson , M. Pinheiro , J. Oliveira , et al., “The Telephysiotherapy for Older People (TOP‐UP) Program for Improving Mobility in People Receiving Aged Care: A Hybrid Type 1 Effectiveness–Implementation Randomised Controlled Trial,” Medical Journal of Australia 223 (2025): 205–213. 40685450 10.5694/mja2.70004 PMC12358105

[8] R. Dawson , H. Gilchrist , M. Pinheiro , et al., “Experiences of Older Adults, Physiotherapists, and Aged Care Staff in the TOP UP Telephysiotherapy Program: Interview Study of the TOP UP Interventions,” JMIR Aging 7 (2024): e53010, 10.2196/53010. 38324369 PMC10882472

[9] A. S. Kebede , L.‐L. Ozolins , H. Holst , and K. Galvin , “Digital Engagement of Older Adults: Scoping Review,” Journal of Medical Internet Research 24, no. 12 (2022): e40192, 10.2196/40192. 36477006 PMC9773036

[10] J. Jimenez , A. Del Rio , A. N. Berman , et al., “Personalizing Digital Health: Adapting Health Technology Systems to Meet the Needs of Different Older Populations,” Health 11, no. 15 (2023): 2140, 10.3390/healthcare11152140. PMC10419052 37570380

[11] H. Edward , N. Constantin , H. Ng , et al., “The Use of Co‐Design in Developing Physical Activity Interventions for Older Adults: A Scoping Review Protocol,” JBI Evidence Synthesis 20, no. 2 (2022): 696–707, 10.11124/jbies-21-00061. 34494611

[12] A. Banbury , S. Pedell , L. Parkinson , and L. Byrne , “Using the Double Diamond Model to Co‐Design a Dementia Caregivers Telehealth Peer Support Program,” Journal of Telemedicine and Telecare 27, no. 10 (2021): 667–673, 10.1177/1357633X211048980. 34726994

[13] Interaction Design Foundation , “What Are Problem Statements?” (2020), https://www.interaction‐design.org/literature/topics/problem‐statements.

[14] P. McBurney , D. Hitchcock , and S. Parsons , “The Eightfold Way of Deliberation Dialogue,” Journal of Intelligent Systems 22, no. 1 (2007): 95–132, 10.1002/int.20191.

[15] M. Hennink and B. N. Kaiser , “Sample Sizes for Saturation in Qualitative Research: A Systematic Review of Empirical Tests,” Social Science & Medicine 292 (2022): 114523, 10.1016/j.socscimed.2021.114523. 34785096

[16] J. Nielsen , “10 Usability Heuristics for User Interface Design,” (2023), https://www.nngroup.com/articles/ten‐usability‐heuristics/.

[17] M. Weber , K.‐U. Schmitt , A. Frei , M. A. Puhan , and A. M. Raab , “Needs Assessment in Community‐Dwelling Older Adults Toward Digital Interventions to Promote Physical Activity: Cross‐Sectional Survey Study,” Digital Health 9 (2023): 20552076231203785, 10.1177/20552076231203785. 37799500 PMC10548814

[18] A. E. Roberts , T. A. Davenport , T. Wong , et al., “Evaluating the Quality and Safety of Health‐Related Apps and e‐Tools: Adapting the Mobile App Rating Scale and Developing a Quality Assurance Protocol,” Internet Interventions 24 (2021): 100379, 10.1016/j.invent.2021.100379. 33777705 PMC7985461

[19] K. S. Pallesen , L. Rogers , S. Anjara , A. de Brún , and E. McAuliffe , “A Qualitative Evaluation of Participants' Experiences of Using Co‐Design to Develop a Collective Leadership Educational Intervention for Health‐Care Teams,” Health Expectations 23, no. 2 (2020): 358–367, 10.1111/hex.13002. 31999883 PMC7104638

[20] V. Braun and V. Clarke , Successful Qualitative Research: A Practical Guide for Beginners (SAGE Publications Ltd, 2013).

[21] R. Chafe , “The Value of Qualitative Description in Health Services and Policy Research,” Health Policy 12, no. 3 (2017): 12–18. PMC5344360 28277201

[22] M. Sandelowski , “Whatever Happened to Qualitative Description?,” Research in Nursing & Health 23 (2000): 334–340. 10940958 10.1002/1098-240x(200008)23:4<334::aid-nur9>3.0.co;2-g

[23] N. K. Gale , G. Heath , E. Cameron , S. Rashid , and S. Redwood , “Using the Framework Method for the Analysis of Qualitative Data in Multi‐Disciplinary Health Research,” BMC Medical Research Methodology 13, no. 1 (2013): 117, 10.1186/1471-2288-13-117. 24047204 PMC3848812

[24] B. Smith and K. R. McGannon , “Developing Rigor in Qualitative Research: Problems and Opportunities Within Sport and Exercise Psychology,” International Review of Sport and Exercise Psychology 11, no. 1 (2018): 101–121, 10.1080/1750984X.2017.1317357.

[25] S. Nordin , A. Davis , F. Légaré , et al., “Designing Meaningful Engagement for Older Adults: An Evaluation of Participation in a Five‐Day Co‐Design Sprint,” Health Expectations 29, no. 1 (2026): e70555, 10.1111/hex.70555. 41521884 PMC12793890

[26] B. S. J. Tay , S. M. Edney , G. D. Brinkworth , et al., “Co‐Design of a Digital Dietary Intervention for Adults at Risk of Type 2 Diabetes,” BMC Public Health 21, no. 1 (2021): 2071, 10.1186/s12889-021-12102-y. 34763701 PMC8582335

[27] Å. Revenäs , C. H. Opava , C. Martin , I. Demmelmaier , C. Keller , and P. Åsenlöf , “Development of a Web‐Based and Mobile App to Support Physical Activity in Individuals With Rheumatoid Arthritis: Results From the Second Step of a Co‐Design Process,” JMIR Research Protocols 4, no. 1 (2015): e22, 10.2196/resprot.3795. 25665589 PMC4342685

[28] V. M. Gallegos‐Rejas , E. E. Thomas , J. T. Kelly , and A. C. Smith , “A Multi‐Stakeholder Approach Is Needed to Reduce the Digital Divide and Encourage Equitable Access to Telehealth,” Journal of Telemedicine and Telecare 29, no. 1 (2023): 73–78, 10.1177/1357633X221107995. 35733379

[29] J. Nurmi , K. Knittle , T. Ginchev , et al., “Engaging Users in the Behavior Change Process With Digitalized Motivational Interviewing and Gamification: Development and Feasibility Testing of the Precious App,” JMIR mHealth and uHealth 8, no. 1 (2020): e12884, 10.2196/12884. 32003750 PMC7055776

[30] V. X. Wu , Y. Chi , J. K. Lee , et al., “The Effect of Dance Interventions on Cognition, Neuroplasticity, Physical Function, Depression, and Quality of Life for Older Adults With Mild Cognitive Impairment: A Systematic Review and Meta‐Analysis,” International Journal of Nursing Studies 122 (2021): 104025, 10.1016/j.ijnurstu.2021.104025. 34298320

[31] A. Windle , A. Marshall , L. de la Perrelle , et al., “Factors That Influence the Implementation of Innovation in Aged Care: A Scoping Review,” JBI Evidence Implementation 22, no. 1 (2024): 61–80, 10.1097/XEB.0000000000000407. 38153118 PMC11163893

[32] C. Benz , W. Scott‐Jeffs , K. A. McKercher , et al., “Community‐Based Participatory‐Research Through Co‐Design: Supporting Collaboration From All Sides of Disability,” Research Involvement and Engagement 10, no. 1 (2024): 47, 10.1186/s40900-024-00573-3. 38730283 PMC11084036

