# Supplementary material for: Co‐Designing a Fall Prevention Exercise Website to Meet Aged Care Community Needs
Source: Australas J Ageing. 2026 Aug 7;45(3):e70216. doi: 10.1111/ajag.70216 (PMC13449867; doi:10.1111/ajag.70216)
Supplement: Supplementary file 1 [file AJAG-45-0-s001.docx]

**Supplementary Material**

**Appendix 1**

**Phase 1 interview guide – Aged Care Consumers: Residential and at home**

Thank you for agreeing to take part in this interview. As you know, we’re looking for ways to improve the program called TOP UP where you have been talking to a physiotherapist using an iPad and doing different types of exercise. We hope that learning more about your experience will help us work out how to make the program easier to deliver and more inviting for people who are taking part in it.

Before we start, I want to let you that I’m audio recording our conversation so that I capture your views accurately, but what you say is confidential which means the research team won’t report any information that could reveal your identity. I’d like to remind you that taking part in this interview is completely voluntary – you are still happy to go ahead with it? Do you have any questions before we start?

As a start, we’d love to hear about your experiences with the TOP UP program (if you can remember back that far):

1. What was it like to take part in the TOP UP physiotherapy program?
2. I’m going to ask you about different parts of the program now. If you don’t remember them it doesn’t matter, just let me know and we’ll skip to the next one.
   a. What was it like getting exercise advice from a physiotherapist over an iPad?
   b. What was it like using an iPad to watch exercise videos?
   c. You had a support worker helping you during the program. What was that like?
   d. Did you keep track of the exercises you were doing? How did you do this?
3. Was there anything you didn’t like about the TOP UP program?
4. What would you change about the TOP UP program if you could?

Now, it would be great to learn more about how well the technology we used for TOP UP works for you:

5. Did you ever use the iPad yourself to access the physiotherapy sessions or the program videos, or did a support worker do that for you? What was that like?
6. [If they did NOT use the tech themselves] Would you *like* to have been able to use the technology yourself? What would have helped you do this?
7. Can we look at the current website together now so I can get your feedback on it? Looking at the website on the iPad now…
   a. Can you find the exercise videos?
   b. How well can you see and hear the exercise videos?
   c. Can you press play/pause/stop on the videos?
   d. Can you read the video introductions?
8. Is there anything you don’t like about using this website? What do you think would make it easier to use?
9. We are thinking of redoing the website so that it is easier to use and more engaging. Do you think it would be helpful if we could add:
a. Zoom or similar teleconference app into the website (so everything is on one place, and you don’t need to use two different software programs)?
b. Exercise videos so they open into large screen format easily?
c. Specific videos demonstrating key exercise like squat, sit to stand for exercise quality training?
d. Videos explaining exercise tips and safety?
e. Getting the website to send you motivational messages or reminders to keep you exercising?

I’d love to hear about any other teledigital health experiences you might have had, by which I mean talking with a health professional over the phone or via video call.

10. Have you done that outside of TOP UP?
11. (If yes to Q21) Is there anything you like about it?
12. (If yes to Q21) Is there anything you don’t like about it?
13. (If yes to Q21) Are there any teledigital health apps or website or other technology you’ve used for this that you can tell us about? What are they like to use?
14. Is there anything else you can tell us that could help us make TOP UP better for older people?

We’re going to develop a website based on your feedback. May we please contact you when we have a draft website ready (in about two months) to ask if you would be interested in testing it and giving us some feedback? It’s completely optional and you are welcome to decline the invitation, at this point we’re just asking if it’s OK for us to contact you and see if you’re interested.

Thank you for your time. Your feedback is really valuable to us.

**Appendix 2**

**Phase 2 Interview guide – Aged Care Consumers**

Introduction

Thank you for helping us test the website we’re developing for the TOP UP program. The website is not finished and at this point we anticipate making many more changes to it so please don’t hold back – any of your thoughts are valuable to us.

Here is the website [provide it on an iPad]. Have a play with it for a few minutes and then we can talk about it.

1. Let’s start with your top-of-mind impressions of the website. What do you think of it?
2. What do you think about the look of the website?
   a. Are the photos easy to see?
   b. Is the text easy to read?
   c. What do you think of the colours?
   d. Can you see how to move around this website?
3. Is there anything you don’t like about the website that we could improve?

Let’s test how easy the website is to use:^[1]^

4. Are you able to access the ‘TOP UP Exercises’ page?
   a. Are the videos easy to locate?
   b. Are you able to press play/pause on the videos?
   c. Are the videos easy to see/watch? Are they big enough?
   d. Is there anything you would change?
5. Please navigate to the *Education* page
   a. What do you think about how the videos are presented?
   b. How easy is it to access and play the videos?
   c. Is there anything you would change?
6. Overall, how easy is the website to use?
   a. How would you rate it from 1-5, one being extremely hard and 5 being very easy?
   b. Did you have any frustrations when using it website?
   c. Is there anything you would change?
   d. Do you have confidence in the information this website provides (e.g. do you confident that it would help you exercise safely and effectively)? Could this be improved?
7. How would you feel about using this website to access physiotherapy and exercise videos?
8. Can you think of anything that’s missing from the website that would help to keep you using it?
9. Have you ever done anything like that before? If so, what was it like?

Thank you for your time. Your feedback is really valuable to us.

1. These functionality questions will be modified and expanded as necessary in order to explore all elements of website, including any customisable features. Given that the website prototype will be co-created in Phase 1 of this study, and may be further developed iteratively during Phase 2, we cannot identify these elements and their intended functionality until the project is underway.
